# Aryl-Boroxazolidones with Low In Vitro Neurotoxicity and Alleviative Effects on MPTP-Induced Parkinsonism in Mice

**DOI:** 10.3390/biom16040494

**Published:** 2026-03-25

**Authors:** Antonio Abad-García, Martiniano Bello, Maricarmen Hernández-Rodríguez, Iris Yuritzi Torres-Deviana, Juan A. García-Guzmán, Karen A. Cruz-Aguayo, Mónica Barrón-González, José G. Trujillo-Ferrara, David Centurion, Marvin A. Soriano-Ursúa

**Affiliations:** 1 Academia de Fisiología, Sección de Estudios de Posgrado e Investigación, Escuela Superior de Medicina, Instituto Politécnico Nacional, Plan de San Luis y Diaz Mirón s/n, Col. Casco de Santo Tomas, Alc. Miguel Hidalgo, Mexico City 11340, Mexico; abadantonio19315@gmail.com (A.A.-G.); mhernandezrod@ipn.mx (M.H.-R.); yuritzidevi29@hotmail.com (I.Y.T.-D.); juangarcia.guz10@gmail.com (J.A.G.-G.); karenare1408@gmail.com (K.A.C.-A.); monicabarronglz@gmail.com (M.B.-G.); 2 Laboratorio de Diseño y Desarrollo de Nuevos Fármacos e Innovación Biotecnológica, Sección de Estudios de Posgrado e Investigación, Escuela Superior de Medicina, Instituto Politécnico Nacional, Plan de San Luis y Diaz Mirón s/n, Col. Casco de Santo Tomas, Alc. Miguel Hidalgo, Mexico City 11340, Mexico; 3 Departamento de Cultivo Celular, Neurofarmacología y Comportamiento, Sección de Estudios de Posgrado e Investigación, Escuela Superior de Medicina, Instituto Politécnico Nacional, Plan de San Luis y Diaz Mirón s/n, Col. Casco de Santo Tomas, Alc. Miguel Hidalgo, Mexico City 11340, Mexico; 4 Laboratorio de Bioquímica, Sección de Estudios de Posgrado e Investigación, Escuela Superior de Medicina, Instituto Politécnico Nacional, Plan de San Luis y Diaz Mirón s/n, Col. Casco de Santo Tomas, Alc. Miguel Hidalgo, Mexico City 11340, Mexico; jtrujillo@ipn.mx; 5 Departamento de Farmacobiología, Centro de Investigación y de Estudios Avanzados, Instituto Politécnico Nacional, Calzada de Los Tenorios 235, Col. Granjas-Coapa, Alc. Tlalpan, Mexico City 14330, Mexico; dcenturi@cinvestav.mx

**Keywords:** Boron, boroxazolidones, Parkinson, neurotoxicity, motor disturbances, serotonin, dopamine receptors

## Abstract

Parkinson’s disease (PD) is one of the most prevalent and extensively studied neurodegenerative conditions. One of its most challenging clinical manifestations is the emergence of dyskinesias, characterized by involuntary movements that significantly impair patients’ quality of life. Meanwhile, boron, as a trace element, and boron-containing compounds have emerged as active modulators of neurotransmitter systems. To evaluate the effect of aryl-boroxazolidones on parkinsonism, the in vitro neurotoxicity of three boroxazolidones was assessed, along with the effects of two of them in mice with parkinsonism induced by MPTP administration. Two novel compounds demonstrated a limitation of parkinsonism, whereas risperidone reduced the beneficial effect of the tested boroxazolidones. The three boroxazolidones did not induce toxicity in neurons or glial cells at concentrations up to 100 µM. In silico analyses support the ability of BCC to act as ligands of dopamine and serotonin receptors. Taken together, these results suggest that the tested boroxazolidones are promising candidate agents, warranting further exploration for the treatment of PD.

## 1. Introduction

Parkinson’s disease (PD) is one of the most prevalent neurodegenerative disorders in the modern world, representing a significant challenge for biomedical research and clinical management. PD is characterized by the progressive loss of dopaminergic neurons in the substantia nigra pars compacta, leading to distinct motor symptoms such as bradykinesia, rigidity, and tremor [1].

Dopamine D2-type receptors (D2DR) are targeted, as they are considered key components in the executive control and working memory of motor behavior. The primary treatment of PD focuses on restoring brain dopamine function; however, it may also, unfortunately, lead to the development of behavioral disorders [2,3,4]. In addition, recent findings have highlighted the importance of serotonin (5-HT) pathways in PD, suggesting that alterations in this system may significantly contribute to a variety of symptoms. The interaction between dopaminergic and serotonergic systems in the brain is complex and multifaceted, implying an interconnection that may be critical for understanding the pathophysiological mechanisms of PD and, consequently, for the development of new therapeutic strategies with fewer adverse effects [5,6]. Several studies have emphasized a crucial role for interactions between serotonergic and dopaminergic systems, mainly in movement control, but also in mood and behavior [6,7,8]. The involvement of both neurotransmitters in motor performance is further supported by the extensive serotonergic innervation of the basal ganglia; additionally, serotonin terminals have been reported to establish synaptic contacts with both substantia nigra dopamine-containing neurons and their terminal regions, such as the striatum, globus pallidus, and subthalamus [9].

On the other hand, boron is recognized as a key trace element in humans, and the incorporation of boron atoms into bioactive chemical structures has led to the development of boron-containing compounds (BCCs), which have recently attracted attention as neuroactive agents due to their activity in the brain [10]. Moreover, a BCC–dopa derivative has recently been reported to alleviate parkinsonism manifestations in MPTP-intoxicated mice, whereas risperidone (a benzoxazole antagonist of dopamine and serotonin receptors [11]) disrupted its effect [12].

In this study, an MPTP-induced murine model of PD was used to evaluate the impact of two aryl-boroxazolidones containing an indole moiety (highly structurally similar to benzoxazole). These compounds had been previously synthesized [12,13,14,15] but had not been tested as neuroactive agents modulating motor activity. In addition, the neurotoxicity of three boroxazolidones was assessed in neuronal and glial cultures. Finally, by combining experimental and computational approaches, this study aims not only to evaluate the efficacy of aryl-boroxazolidones in improving motor symptoms in a PD model, but also to investigate their potential interactions with dopaminergic and serotonin receptors, particularly in light of the observation that risperidone, an antagonist with affinity for these receptors, disrupts their in vivo effects.

## 2. Materials and Methods

### 2.1. Chemical Reagents and Drugs

**Figure 1:**
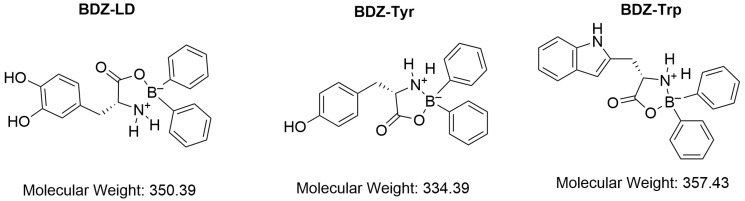
Structures of the boroxazolidone compounds evaluated in this study.

The synthesis of the three boroxazolidones (Figure 1) was carried out through the reaction of the aromatic α-amino acids L-tyrosine (CAS 60-18-4; Sigma-Aldrich, St. Louis, MO, USA), L-tryptophan (CAS 73-22-3; Sigma-Aldrich, St. Louis, MO, USA), or levodopa (CAS 59-92-7; Sigma-Aldrich, Burlington, MA, USA) with 2-amino-diphenyl borinate (2-APB) (CAS 524-95-8; Sigma-Aldrich, St. Louis, MO, USA) in a hydrochloric acid solution under reflux for three hours using anhydrous ether. The crude reaction product was allowed to stand on filter paper for 48 to 72 h, yielding a white solid with a cotton-like consistency, as previously reported [12]. Risperidone was used as supplied (CAS 106266-06-2; Sigma-Aldrich, St. Louis, MO, USA).

Boroxazolidones were synthesized and appropriately characterized by comparison with previously reported data. Analyses were performed using melting point determination, RF values in silica thin-layer chromatography, infrared (IR), and nuclear magnetic resonance (NMR) spectroscopy. A characteristic signal corresponding to the tetracoordinated boron atom at δ 5.2–7.5 ppm was identified for each compound [12,13,14,15].

### 2.2. Evaluation of Cytotoxicity in Primary Astrocyte Cell Culture

To isolate neonatal rat astrocytes, cerebral hemispheres from 1–2-day-old Wistar rats were surgically removed under aseptic conditions and placed in Dulbecco’s Modified Eagle Medium (DMEM) (Gibco, Thermo Fisher Scientific, Waltham, MA, USA). Subsequently, the meninges were carefully removed, and the brain tissue was minced and mechanically dissociated for 3–5 min. The resulting mixed astroglial cell suspension was plated in 75 cm^2^ vented culture flasks containing DMEM supplemented with 10% fetal bovine serum (FBS) (Gibco, Thermo Fisher Scientific, Waltham, MA, USA), 120 U/mL penicillin, and 12 µg/mL streptomycin (Gibco, Thermo Fisher Scientific, Waltham, MA, USA), and maintained in a humidified incubator with 5% CO_2_ at 37 °C for 7 days. On day 8, microglial cells were removed using an orbital shaker at 150 rpm for 1 h. The enriched astrocyte culture remaining attached to the flask was then maintained under the previously described conditions until confluence was reached [16].

Astrocytes were detached from the culture flasks by adding 3 mL of 0.25% trypsin–EDTA (GIBCO Laboratories) and subsequently diluted with fresh medium. Cell numbers were determined by trypan blue exclusion using an automated cell counter (Thermo Fisher Countess 3 FL). A cell suspension (100 μL containing 50,000 cells) was seeded into 96-well culture plates and incubated for 24 h at 37 °C in a 5% CO_2_ atmosphere. Subsequently, 100 μL of solutions containing increasing concentrations of the studied compounds, diluted in fresh medium with 2% DMSO (Sigma-Aldrich, Burlington, MA, USA), were added to the respective wells to achieve final concentrations of 6.25, 12.5, 25, 50, 100, and 200 μM (*n* = 8), followed by incubation for an additional 24 h (final DMSO concentration: 1%). Cells cultured in medium containing 2% DMSO were used as controls.

Thereafter, 20 μL of MTT reagent (5 mg/mL) (Invitrogen, Thermo Fisher Scientific, Waltham, MA, USA) was added to each well, and the plates were incubated for an additional 4 h. At the end of the incubation period, the culture medium was carefully removed, and the formazan crystals produced by viable cells were solubilized by adding 150 μL of 4 mM HCl in isopropanol. Absorbance was measured at 540 nm using a UV/Vis spectrophotometer (Emax Precision microplate reader, Molecular Devices, San Jose, CA, USA). Absorbance values from untreated control cells were considered as 100% viability. All assays were performed in triplicate. Concentration–response curves were generated for the evaluated compounds, and the half-maximal inhibitory concentration (IC_50_) was calculated by linear regression analysis.

### 2.3. Evaluation of Cytotoxicity in Primary Hippocampal Neuronal Cell Culture

Full-term pregnant Wistar rats were obtained from the Animal Facility of the Institute of Cellular Physiology at UNAM and maintained in polycarbonate cages under standard conditions (12:12 h light/dark cycle, stress-free environment, water ad libitum, and standard diet) at the Laboratorio de Cultivo Celular, Neurofarmacología y Conducta of the Escuela Superior de Medicina. After birth, newborn rats were left in the care of their mothers. For this procedure, neonatal Wistar rat pups of both sexes, aged 1 to 5 days, were used. Briefly, pups were rinsed with 70% ethanol and decapitated.

Cells for primary cultures were obtained from the telencephalon of Wistar rat embryos at 18 days of gestation (E18). Pregnant rats were anesthetized with ethyl ether prior to the extraction of the uterine horns. Embryos were transferred under sterile conditions into Falcon tubes containing cold, sterile Hanks’ Balanced Salt Solution (HBSS) (Gibco, Thermo Fisher Scientific, Waltham, MA, USA). The embryos were decapitated, and the heads were placed in HBSS. Brains were carefully removed, and the hippocampi were dissected under a stereoscopic microscope, discarding any tissues damaged during extraction. The tissues were washed ten times with sterile HBSS and subjected to enzymatic dissociation by incubation with 2.5% trypsin/EDTA (Gibco, Thermo Fisher Scientific, Waltham, MA, USA) for 15 min at 37 °C without agitation. Subsequently, tissues were washed with DMEM supplemented with 10% FBS.

The tissues were then transferred to sterile 15 mL Falcon tubes containing 2 mL of DMEM and mechanically dissociated using a blunt-tipped glass Pasteur pipette. Once a homogeneous cell suspension was obtained, cell numbers were determined by trypan blue exclusion using an automated cell counter (Thermo Fisher Countess 3 FL). Cells were seeded into 24-well plates coated with poly-L-lysine (prepared the previous day using 0.01% *w*/*v* poly-L-lysine hydrobromide; Sigma-Aldrich, Burlington, MA, USA) at a density of 128,000 cells per well and maintained at 37 °C in a 5% CO_2_ atmosphere. One hour after plating, the DMEM was replaced with Neurobasal medium (Gibco, Thermo Fisher Scientific, Waltham, MA, USA) supplemented with 2% B27, 0.5 mM L-glutamine, and 1% (*v*/*v*) penicillin–streptomycin (all from Gibco, Thermo Fisher Scientific, Waltham, MA, USA). Cultures were maintained for 7 days, with medium changes every 2–3 days by replacing 20% of the medium with fresh Neurobasal. This time point corresponds to mature neuronal cultures.

On day 8, 100 μL of solutions containing increasing concentrations of the studied compounds, diluted in fresh medium with 2% DMSO, were added to the respective wells to achieve final concentrations of 25, 50, and 100 μM (*n* = 6), followed by incubation for an additional 24 h (final DMSO concentration: 1%). Subsequently, 20 μL of MTT reagent (5 mg/mL) was added to each well, and the plates were incubated for an additional 4 h. At the end of the incubation period, the culture medium was carefully removed, and the formazan produced by viable cells was solubilized by adding 150 μL of 4 mM HCl in isopropanol. Absorbance was measured at 540 nm using a UV/Vis spectrophotometer (Emax Precision microplate reader, Molecular Devices, San Jose, CA, USA). Absorbance values from untreated cells were considered as 100% viability. All assays were performed in triplicate. Concentration–response curves were generated for the evaluated compounds, and the half-maximal inhibitory concentration (IC_50_) was calculated using linear regression analysis.

### 2.4. Motor Evaluation in MPTP-Intoxicated Mice

Six groups of eight randomly distributed male C57BL/6 mice (25 ± 2 g, eight weeks old) were used to evaluate the effects of 1-methyl-4-phenyl-1,2,3,6-tetrahydropyridine (MPTP; CAS 23007-85-4, Sigma-Aldrich), along with an additional four groups to assess the effect of risperidone (total *n* = 80). Animals were housed in 70 × 40 × 35 cm cages under controlled conditions, with room temperatures ranging from 12 to 20 °C, and had free access to food and water throughout the experimental period. To induce an acute intoxication model, the neurotoxin MPTP was dissolved in distilled water and administered intraperitoneally (i.p.) at a dose of 18 mg/kg, four times at 2 h intervals, as previously described for parkinsonism induction [12].

Two hours after MPTP administration, behavioral evaluations were conducted using open field, rotarod, and grasp tests under low-light conditions to minimize stress and environmental variability. These tests assess locomotion, coordination, balance, muscle strength, and fine motor skills.

Treatments were administered intraperitoneally as follows: group 1 (control) received saline solution twice; groups 2–6 were subjected to MPTP intoxication. Group 2 received saline as treatment, group 3 received levodopa at a dose of 50 mg/kg, and groups 4–6 received boroxazolidones derived from levodopa (BDZ-LD), L-tyrosine (BDZ-Tyr), and L-tryptophan (BDZ-Trp), respectively, at equimolar doses relative to the standard levodopa treatment. Evaluations were performed 30 min after treatment administration and repeated daily for the following three days. For groups 7–10, treatments were administered as in groups 1 and 4–6, with prior administration of risperidone at a dose of 2 mg/kg via the same route, as previously reported [12]. All solutions were prepared for intraperitoneal administration at a volume of 10 mL/kg.

This study was conducted in accordance with the Official Mexican Standard NOM-062-ZOO-1999, which establishes technical specifications for the production, care, and use of laboratory animals. All procedures were approved by the Biosafety Committee of CBS-ESM-IPN (protocol ESM-CBS-03/8-10-2018) and the Institutional Committee for the Care and Use of Laboratory Animals (CICUAL-06/26–08-2019). C57BL/6 mice were obtained from the Vivarium of the Universidad Autónoma del Estado de Hidalgo (UAEH).

### 2.5. Docking Assays

To explore potential interactions between the tested BCC and receptors involved in the attenuation of parkinsonism, docking assays were performed using BCC as ligands against D2DR and 5-HT2 receptors. The binding site, ligand pose, as well as the estimated free energy and affinity (expressed as pKi) were analyzed.

The D2DR model employed was retrieved, prepared, and structurally evaluated as previously described [12]. Briefly, the D2DR model was obtained from the Protein Data Bank (PDB ID: 9BS9), a refined LSD-bound structure widely used for the analysis of ligands targeting both D2DR and 5-HT2A receptors [17]. For the 5-HT2A receptor, the model used corresponded to the structure crystallized with risperidone (PDB ID: 6A93), as this ligand is well known to bind both D2DR and 5-HT2A receptors [18]. Ligands were removed from the complexes, and receptor structures were prepared for docking as previously described [12].

Boroxazolidones were constructed as ligands using the GaussView 5 graphical interface [19]. Ligand structures were modeled at pH 7.4, and geometric optimization was performed to determine the most stable conformer of each molecule at a semi-empirical level using the AM1 method. Vibrational frequency calculations were carried out using Gaussian 09 [20] to confirm that the optimized conformations corresponded to minimum energy states. Subsequently, ligands were prepared following a standard docking protocol, as described elsewhere [12]. Docking results were visualized using VMD software (v1.9.6; Theoretical and Computational Biophysics Group, Urbana, IL, USA) [21], and free energy and Ki values were obtained from the AutoDock program (v4.2.6; The Scripps Research Institute, La Jolla, CA, USA) [22].

### 2.6. Statistics

Regarding statistical analysis, tests such as ANOVA for multiple comparisons were performed following a Shapiro–Wilk test for normality, and were followed by post hoc Tukey’s tests when appropriate. Results are presented as mean ± standard error of the mean. All analyses were conducted using GraphPad Prism statistical software (v.10.0; Boston, MA, USA), with the significance level set at *p* < 0.05.

## 3. Results

### 3.1. In Vitro Toxicity Evaluation

#### 3.1.1. Astrocytes

**Figure 2:**
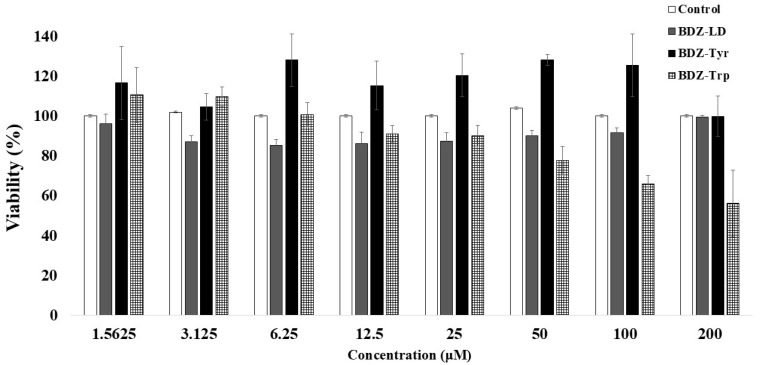
Quantitative assessment of the cytotoxic effects of selected compounds on primary astrocytic cell cultures. A total of 50,000 astrocytic cells per well were incubated in the absence or presence of increasing concentrations of the compounds for 24 h. Subsequently, cells were incubated with MTT reagent for 4 h, after which absorbance was measured at 540 nm, and cell viability was calculated. Values shown in the graph represent mean ± standard error (*n* = 8). No significant differences were observed among groups (*p* ≥0.0612).

Astrocyte cells were incubated with varying concentrations of the studied compounds for 24 h to determine cell viability by measuring cytoplasmic dehydrogenase activity. The results are shown in Figure 2. As observed, only BDZ-Trp exhibited cytotoxicity in astrocytes, but with an IC_50_ > 200 µM.

In addition, cell viability of 99.9% and 99.3% was observed at a concentration of 200 µM for compounds BDZ-Tyr and BDZ-LD, respectively, thereby evidencing their low cytotoxicity in astrocytic cell cultures.

#### 3.1.2. Neurons

**Figure 3:**
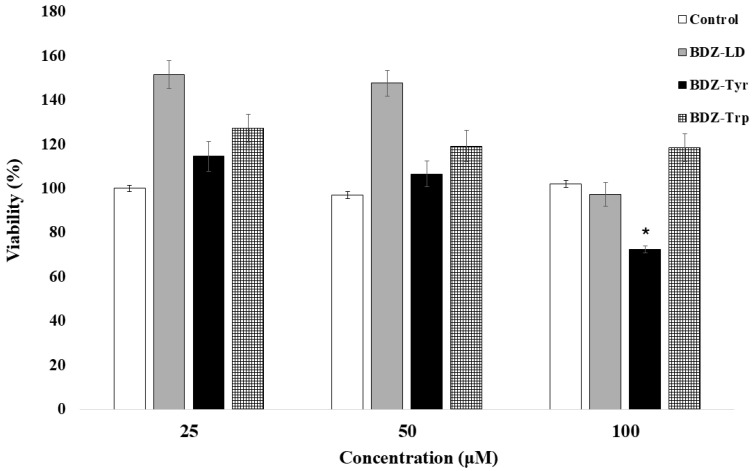
Quantitative assessment of the cytotoxic effects of selected compounds on primary neuronal cell cultures. A total of 128,000 cells per well were incubated either in the absence of compounds or with increasing concentrations of the compounds for 24 h. Subsequently, cells were incubated with MTT reagent for 4 h, after which absorbance was measured at 540 nm, and cell viability was calculated. Values shown in the graph represent mean ± standard error (*n* = 6). An asterisk indicates a significant difference compared to the control group (*p* = 0.0024).

The results of the cytotoxicity studies in neurons are shown in Figure 3, indicating that only BDZ-Tyr caused a minimal decrease in cell viability at 100 µM (72.3% for BDZ-Tyr).

### 3.2. In Vivo

#### 3.2.1. Motor Behavior

**Figure 4:**
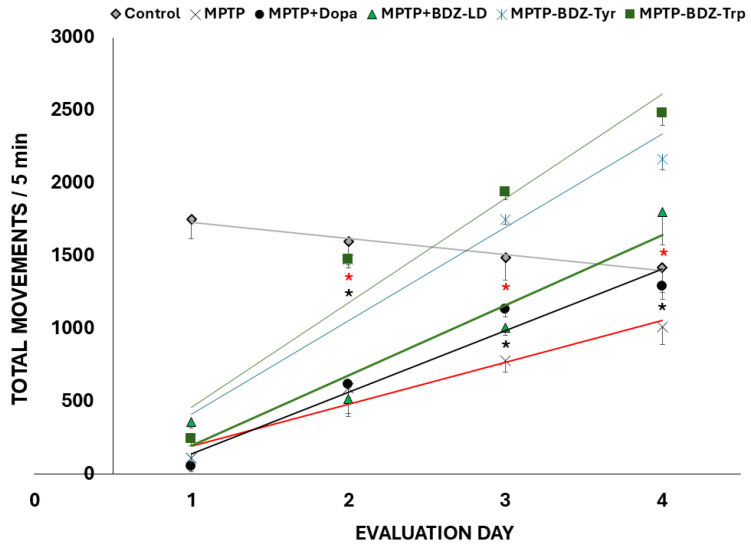
Effects on performance in the open field test. Dopa: levodopa. Values are presented as mean ± standard error of the mean (*n* = 8). Black asterisks indicate groups with values significantly different from the MPTP group, whereas red asterisks indicate groups with values significantly different from the MPTP + Dopa group (*p* ≤ 0.05).

Performance in the open field test was markedly reduced following MPTP administration (Figure 4). However, levodopa administration alleviated this impairment, with significant effects observed on the third and fourth days of evaluation. Similarly, the administration of any of the boroxazolidones limited the reduction in movement induced by MPTP. Moreover, mice treated with BDZ-Tyr and BDZ-Trp exhibited increased locomotor activity from the second to the fourth day of evaluation compared to those treated with levodopa or BDZ-LD.

Results from the rotarod and grasp tests were consistent with the observed improvements in motor function in MPTP-intoxicated mice (Supplementary Figures S1 and S2). In the rotarod test, performance improved in all treated groups after the first day of evaluation, whereas in the grasp test, improvement was clearly observed after the second day. No differences in the degree of improvement were observed among the boroxazolidones in these tests.

#### 3.2.2. Effect of Risperidone on the Induced Effect by Boroxazolidones

**Figure 5:**
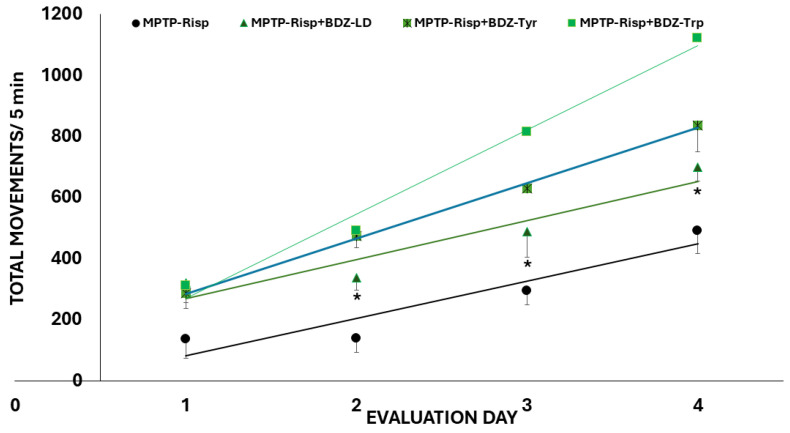
Effects of risperidone on performance in the open-field test. Risp: risperidone. Values are presented as mean ± standard error of the mean (*n* = 8). Asterisks indicate groups with values significantly different from the MPTP–Risp group (*p* ≤ 0.0482).

The ameliorative effect of boroxazolidones on MPTP-induced motor impairment was modified by the administration of risperidone. Indeed, improvement—reflected by increased movement—was still observed; however, the maximum number of movements (previously observed after boroxazolidone administration) was approximately half of that observed in the absence of risperidone. Notably, the effect of risperidone appeared to follow the order BDZ-LD > BDZ-Tyr > BDZ-Trp (Figure 5).

### 3.3. In Silico

**Figure 6:**
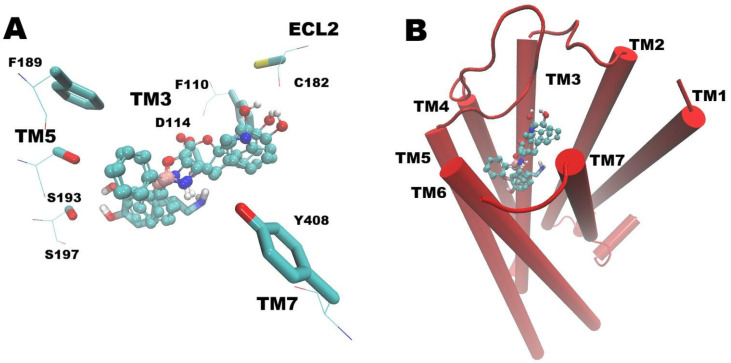
Docking of dopamine and the three tested BCC on the D2 dopamine receptor (PDB ID: 9BS9). Dopamine is represented as transparent sticks, whereas all three BCC are shown in CPK representation, with atoms colored by type: carbon in cyan, oxygen in red, nitrogen in blue, and hydrogen in white. (**A**) Extracellular view highlighting key residues involved in ligand interactions (residues forming the toggle switch in TM6 were omitted for clarity). (**B**) Visualization of the entire receptor and the localization of the ligand-binding pocket. TM = transmembrane domain; ECL2 = second extracellular loop.

**Figure 7:**
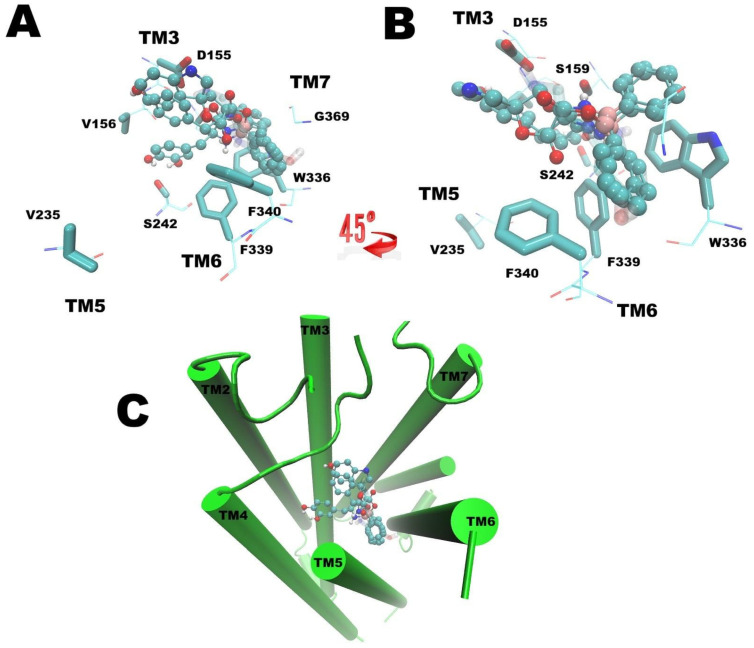
Docking of serotonin and the three tested BCC on the 5-HT2A serotonin receptor (PDB ID: 6A93). Serotonin is represented as transparent sticks, whereas all three BCC are shown in CPK representation, with atoms colored by type: carbon in cyan, oxygen in red, nitrogen in blue, and hydrogen in white. (**A**) Extracellular view highlighting key residues involved in ligand interactions; the catechol moiety in the BDZ-LD compound is clearly oriented toward S242 in TM5, whereas the other compounds are directed toward a shallower region than the dopamine binding site. (**B**) Additional extracellular view showing the overlap of boron atoms and phenyl moieties toward the toggle switch in TM6. (**C**) Visualization of the entire receptor and the localization of the ligand-binding pocket. TM = transmembrane domain.

All docked compounds exhibited interactions within the canonical binding pocket of dopamine and serotonin receptors, consistent with previously reported binding models (Figure 6 and Figure 7) [17,18]. Notably, the presence of boron was associated with higher predicted affinities, as reflected by the pKi values summarized in Table 1.

Boroxazolidones were found to dock within the dopamine binding pocket of D2DR. The diphenyl moiety consistently projected toward the region where dopamine binds, whereas the substituents attached to the boron atom (phenol, catechol, or indole) extended toward a more superficial subpocket adjacent to the main cavity.

In the 5-HT2A receptor, the tested boroxazolidones occupied the cavity where risperidone and LSD interact. All compounds positioned the boron atom in a comparable location, with the diphenyl moiety oriented toward the subpocket formed by transmembrane helices (TM) 3, 6, and 7. For BDZ-Tyr and BDZ-Trp, the phenolic and indole moieties, respectively, projected into a shallow, expanded region of the pocket, whereas in BDZ-LD, the catechol group was oriented toward the conserved serine residues of TM5.

## 4. Discussion

PD remains one of the major neurological disorders with a high global burden, and projections suggest that its impact may continue to increase in the coming years [23,24].

Pharmacological treatment of PD is primarily centered on restoring dopamine transmission in the central nervous system. Administration of levodopa (or dopamine receptor agonists) remains the main therapeutic strategy. Furthermore, dopamine exerts pro-locomotor effects through activation of D2 receptors, which decreases the excitability of neurons in the indirect pathway. These basal ganglia circuits are known to contribute to increased locomotor activity induced by psychostimulants, such as amphetamines or cocaine, which enhance dopamine release [25,26]. Unlike dopamine agonists, levodopa acts on both families of dopamine receptors and may also influence other amine receptor systems, including noradrenaline and serotonin receptors, thereby functioning as a neuromodulator [1,26].

However, the identification of multiple non-dopaminergic pathways involved in motor control and basal ganglia function has attracted considerable interest for the development of new therapeutic strategies for PD [27,28,29]. Among additional neurotransmitters of interest for motor modulation, serotonin and its receptors have gained particular attention. Initially, the interaction between catecholaminergic and serotonergic receptors was studied in the context of emotional regulation [30]. Given the involvement of the serotonergic system in PD, it is noteworthy that Lewy bodies are detected early in raphe neurons during disease progression, and that PD is characterized by limbic symptoms such as anxiety and depression, which may influence motor performance in murine models [30,31]. More recently, attention has expanded to include both motor and non-motor manifestations [31]. Moreover, 5-HT2A receptor agonists have been shown to improve motor performance in MPTP-treated mice [32], and depletion of both dopamine and serotonin in MPTP-intoxicated mice has been implicated in motor deficits [5,33].

Specifically, the 5-HT2A receptor appears to be a key serotonergic component involved in the regulation of basal ganglia activity [32,34,35]. In parallel, levodopa administration in patients with PD has been associated with alterations in the serotonergic system, likely involving this receptor as a major mediator [35,36]. In addition, serotonin receptor expression and activity are critical for motor control, mood regulation, and gastrointestinal function in both physiological and pathological conditions. In patients with PD, serotonergic transmission is reduced in advanced stages, with decreased levels of serotonin and its metabolite 5-hydroxyindoleacetic acid (5-HIAA), as well as reduced expression of the serotonin transporter (SERT) in several basal ganglia nuclei. Therefore, the interaction between dopaminergic and serotonergic systems is a key factor in the development of novel therapeutic approaches for PD.

On the other hand, boron-containing compounds (BCCs) have emerged as pharmacologically relevant agents, and their biological activity is increasingly being characterized [37,38]. In particular, boroxazolidones have been synthesized and reported as safe and biologically active compounds [14,39,40,41,42], including evidence of activity within the mammalian nervous system [10,15,43]. Additional BCCs have demonstrated effects in glial tumors, notably glioblastoma [42], and several organoboron derivatives of α-amino acids have been shown to modulate mammalian behavior [13,37].

To our knowledge, this is the first study evaluating the cytotoxicity of boroxazolidones in primary rat brain cells. The compounds exhibited no detectable cytotoxicity at concentrations up to 100 µM in neurons and up to 200 µM in astrocytes, exceeding those expected to be reached in the brain during pharmacological activity. These findings are consistent with previous reports describing low toxicity of boronic acids or boronates in neuron-related cells (tested up to 300 µM in PC12 cells, derived from an adrenal pheochromocytoma) [43], as well as boron-containing carbohydrate derivatives in primary rat neurons (100–200 µM) [16].

Regarding their pharmacological activity, as suggested by the behavioral evaluation of MPTP-intoxicated mice pretreated with risperidone, these compounds may offer the potential not only to restore dopaminergic balance but also to interact with serotonergic pathways, thereby providing a more comprehensive approach to PD treatment. The ability of aryl-boroxazolidones to modulate these neurochemical systems could represent a significant advancement in PD therapy, addressing a broader spectrum of symptoms and potentially influencing disease progression.

Notably, the effects observed following prior administration of risperidone (an antagonist of D2DR and 5-HT2A receptors, with nanomolar affinity for these targets, with Ki values of 3.2 and 0.2 nM, respectively, but micromolar affinity for other related receptors) differed among groups treated with distinct boroxazolidones. The attenuating effect of risperidone was most pronounced in mice treated with the levodopa-derived compound (BDZ-LD). In contrast, mice treated with BDZ-Tyr and BDZ-Trp appeared less affected; although pretreatment with risperidone still resulted in improved motor performance, this did not reach the level observed in control animals, but rather resembled that of partially recovered mice by the fourth day after MPTP administration (Figure 4 and Figure 5). Differences were also evident across parameters such as overall motor activity, coordination, and strength, reflecting the specific domains assessed by the behavioral tests. These observations may suggest the involvement of additional mechanisms of action, particularly for BDZ-Tyr and BDZ-Trp. Further studies, including pharmacological evaluations in systems expressing the targeted receptors, are required to establish a structure–activity relationship; however, differences in the substituent moieties attached to the five-membered ring may contribute to the observed biological effects, given that the core structure is shared among the compounds.

Additionally, the in silico findings provide relevant insights. First, they support coherent interactions with both receptors potentially involved. Second, they highlight differences in receptor interactions among compounds. In most docking poses, the variable moieties were directed toward a shallower region of the binding pocket; however, BDZ-LD displayed a distinct interaction pattern at the 5-HT2A receptor. This orientation resembles that of catecholamines interacting within dopamine or adrenergic receptor binding sites, where the catechol moiety is directed toward conserved serine residues in transmembrane domain 5 (e.g., Ser242, as illustrated in Figure 7), which is also conserved in serotonin receptors (position 5.46 according to Ballesteros–Weinstein nomenclature). In contrast, BDZ-Tyr and BDZ-Trp docked in a manner in which their distinct moieties were oriented toward a secondary binding pocket associated with functional selectivity in aminergic receptors [44,45,46,47]. It has been proposed that interactions within this secondary pocket contribute significantly to ligand affinity, selectivity, functional activity, signaling bias, and binding kinetics, and that targeting this region may enable the design of ligands with tailored pharmacological profiles [47].

Accordingly, recent studies have described ligands capable of interacting with dopamine and serotonin receptors beyond the orthosteric site, including engagement with shallower secondary pockets [17,46,48,49]. Such mechanisms may contribute to explaining both the in vivo and in silico findings observed for the tested boroxazolidones, although additional experimental evidence is required to confirm or refute a potential bitopic binding mode. It should also be noted that the boron atom itself does not appear to directly interact with receptor residues; however, its electronic properties and the chemical environment provided by its substituents may play a critical role in positioning the ligand near key residues involved in receptor activation, such as those at position 3.32 (D114 in D2DR and D155 in 5-HT2A).

Overall, the present results indicate that aryl-boroxazolidones do not exhibit initial signs of cytotoxicity, as assessed by MTT assays in primary rat brain cell cultures, and are capable of alleviating MPTP-induced parkinsonism. Nevertheless, further investigation is required, as current findings provide only preliminary insights into their mechanism of action. Additional studies addressing neuronal damage at the morphological level, as well as pharmacokinetic profiling following intraperitoneal administration, are warranted [50]. In this context, emerging models such as single-neuron degeneration may prove useful for future evaluation. This model is based on aminochrome-induced neurotoxicity, which involves mitochondrial dysfunction, formation of neurotoxic oligomers, impairment of lysosomal and proteasomal degradation systems, endoplasmic reticulum stress, neuroinflammation, and oxidative stress, ultimately leading to the loss of individual neuromelanin-containing dopaminergic neurons without affecting neighboring cells [51]. Furthermore, such approaches may help identify key signaling pathways involved in the neuroprotective effects of boroxazolidones; for example, modulation of the KEAP1/NRF2 signaling pathway has been recognized as critical in this model [52].

Despite these considerations, the development of compounds targeting both dopaminergic and serotonergic receptors remains an attractive strategy, given their potential applications beyond motor disorders. These include psychiatric conditions and other diseases currently under clinical investigation, such as diabetic retinopathy, multiple sclerosis, non-functioning pituitary adenomas, alcohol use disorder, anorexia, delirium, post-traumatic stress disorder, depression, and epilepsy [49].

Finally, it is important to acknowledge the inherent limitations of acute intoxication models of PD in mice and their extrapolation to human disease [53], as well as the potential influence of genetic and environmental variability on experimental outcomes [53,54]. Additionally, the relatively low rate of successful translation from preclinical studies to clinical application highlights the need for further validation. A major limitation of exogenous neurotoxin models such as MPTP is the induction of a rapid neurodegenerative process, which contrasts with the progressive nature of Parkinson’s disease [55]. Additionally, a characteristic and limitation of this study is the use of risperidone, a compound that acts on both dopamine and serotonin receptors. The use of more selective compounds across different experimental systems—including cells, tissues, organs, or whole organisms—would allow for the assessment of potential selective or biased activity at specific receptors. Furthermore, the limited knowledge regarding the pharmacokinetic profile of boroxazolidones should be considered a limitation, as their effects should be evaluated using different dosages and routes of administration. Finally, in the in silico analyses, docking was performed under static conditions for selected receptors; however, evaluating the dynamic behavior of receptors in the presence of BCC ligands would be valuable for identifying key interactions and conformational changes associated with receptor activity and signaling.

## 5. Conclusions

The present results show that all three tested boroxazolidones (derived from levodopa, tyrosine, and tryptophan) do not exhibit cytotoxicity in primary rat brain cell cultures (glial and neuronal) and act in a time- and concentration-dependent manner on parkinsonism induced by MPTP administration. Further studies are required to elucidate their mechanism of action; however, in silico analyses and the attenuation of their effects by risperidone suggest that these compounds interact with dopamine and/or serotonin receptors to produce effects relevant to PD treatment.

Additionally, further investigations addressing morphological changes, neuroprotective effects associated with the limitation of neuronal loss in relevant nuclei, and the identification of key proteins, pathways, metabolites, reactive species, and genes in mice subjected to induced parkinsonism and treatment are warranted to expand the understanding of the findings reported in this work.

## Figures and Tables

**Table 1:** Predicted affinity of some ligands on D2DR and 5-HT2A receptors.

	Calculated pKi on	
Ligand	D2DR	5-HT2A
Dopamine	4.5063	4.2381
Levodopa	4.5356	4.0542
Tyrosine	4.7231	4.0928
Tryptophan	5.1752	5.0862
Serotonin	5.3947	5.2358
Risperidone	5.8584	5.4245
BDZ-Tyr	6.0381	6.3102
BDZ-LD	6.2135	6.4101
BDZ-Trp	7.3555	7.1518

## Data Availability

Any additional data to those presented in this manuscript can be requested by e-mail to corresponding authors.

## Supplementary Materials

The following supporting information can be downloaded at https://www.mdpi.com/article/10.3390/biom16040494/s1, Supplementary Information: Data for chemical characterization of used boroxazolidones. Figure S1: Effects on the performance in the rotarod test; Figure S2: Effects on the performance in the open field test.
