# Supplementary material for: Aryl-Boroxazolidones with Low In Vitro Neurotoxicity and Alleviative Effects on MPTP-Induced Parkinsonism in Mice
Source: Biomolecules. 2026 Mar 25;16(4):494. doi: 10.3390/biom16040494 (PMC13113705; doi:10.3390/biom16040494)
Supplement: Supplementary file 1 [file biomolecules-16-00494-s001.zip › biomolecules-4180301-supplementary.pdf]

Supplementary Information

Data for chemical characterization of used boroxazolidones:

BDZ-LD: **m.p.:** 264–266 °C; **IR vmax (cm−1)**: 1521.4, 1569, 1602.6, 1726.37, 3227, 3285.8, 3535. **^{1}H NMR** (400 MHz; MeOD) δ 7.47-7.11 (10H, m, H-8:12 and 14:18), 6.72 (1H, d, *Jm* _2.1 Hz, H-2_), 6.67 (1H, d, *Jo* _8.0 Hz, H-5_), 6.51 (1H, dd, *Jo* _8.0 Hz and *Jm*_1.8 Hz, H-6_), 3.88 (1H, m, H-4), 2.98 (2H, m, H-6). **^{13}C NMR** (101 MHz; MeOD) δ 175.45 (COOR, C-5), 145.67 (C, *ipso*), 144.70 (C, *ipso*), 131.05 (C, C-1_), 126.98 (CH,*o*), 120.61 (CH, *m*), 115.87 (CH, *p*), 56.68 (CH, C-4), 34.45 (CH2, C-6). **^{11}B NMR** (128 MHz; MeOD) δ 7.44.

BDZ-Tyr: **m.p**.: 149-150°C; **IR vmax (cm−1)**: 1327.2, 1437.1, 1736.0, 2937, 3076. **^{1}H NMR** (300 MHz; DMSO-d6) 6 9.34 (b, lH, OH), 7.43, and 7.41 (2 d, ***J*** = 7 Hz, 4H, H ortho), 7.22 (t, *J* = 7 Hz, 2H, H *mefa),* 7.20 (t, *J* = 7 Hz, 2H, H *meta'),* 7.17 (t, *J* = 7 Hz, lH, H para), 7.14 (t, *J* = 7 Hz, lH, H para'), 7.06 (d, *J* = 8 Hz, 8 Hz, 1H. NH), 3.68-3.54 (m, lH, H-2), 3.06 (dd, *J* = 14, 3 Hz, lH, H-3), and 2.83 (dd,J = 14, 10 Hz,2H, H-5), 6.72 (d, *J* P 8 Hz, 2H, H-6), 6.64 (t, *J* = lH, H-3'). **^{13}C NMR** (101 MHz; MeOD) δ 173.70 (COOR, C-5), 147.70 (C, *ipso*), 147.30 (C, *ipso*), 13.05 (C, C-1_), 126.98 (CH,*o*), 127.10 (CH, *m*), 125.90 (CH, *p*), 57.30 (CH, C-4), 37.50 (CH2, C-6). **^{11}B NMR** (128 MHz; MeOD) δ 3.00.

BDZ-Trp: **m.p.**: 260–262 °C; **IR vmax (cm−1)**: 424, 706, 748, 971, 1432, 1596, 1710, 3411. **^{1}H NMR** (300 MHz; D6-DMSO) _ 9.23 (1H,s, H7-indole), 7.33 (4H, ddt, J = 8.1, 6.3, 1.5, H10-13), 7.26–6.93, 10H, m, H17-21,H170-210), 6.67–6.51 (3H, m, H4, H3-ammonium), 3.52 (2H, J = 8.5 Hz, H7-indole), 2.98 (1H, dd, J = 14.4, 3.9 Hz, H6), 2.73 (1H, dd, J = 14.5, 10.3 Hz, H60). **^{13}C NMR** (75 MHz; D6-DMSO) _ 174.01 (COOR, C5), 156.61 (C16, ipso), 131.57 (C14), 131.23 (CH, o), 130.66 (CH, C15), 127.52 (CH, m), 127.44 (CH, p), 127.37 (CH, C12), 126.37(CH, C11), 126.28(CH, C10), 115.68(CH, C9), 57.47 (CH, C4), 34.59 (CH2, C6). **^{11}B NMR** (128 MHz; MeOD) δ 5.26.

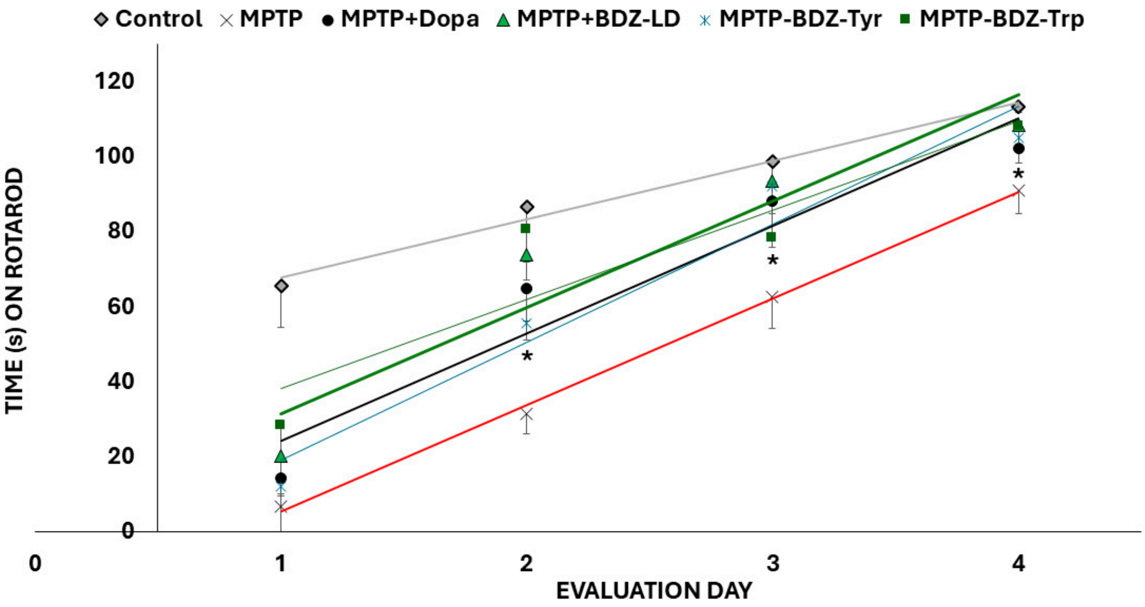

Figure S1. Effects on the performance in the rotarod test. Dopa: Levodopa. Mean values ± standard error mean, n=8. Black asterisks are below the groups with values different against the MPTP group ($p \leq 0.05$).

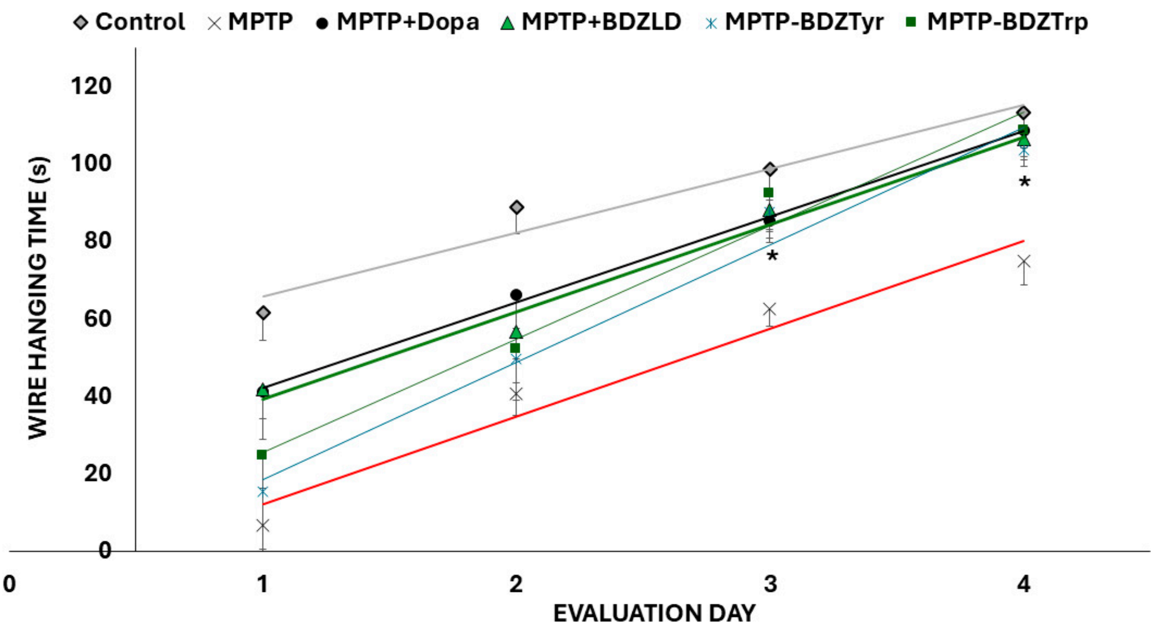

Figure S2. Effects on the performance in the open field test. Dopa: Levodopa. Mean values ± standard error mean, n=8. Black asterisks are below the groups with values different from the MPTP group (p ≤ 0.05).
